# A51 MICROBIALLY-MEDIATED IMPAIRMENT OF VAGAL AFFERENT NEURONAL EXCITABILITY IS OREXIN RECEPTOR DEPENDENT

**DOI:** 10.1093/jcag/gwad061.051

**Published:** 2024-02-14

**Authors:** S Sachdev, A Tashtush, T Alward, D E Reed, A E Lomax

**Affiliations:** Biomedical and Molecular Sciences, Queen's University, Kingston, ON, Canada; Biomedical and Molecular Sciences, Queen's University, Kingston, ON, Canada; Biomedical and Molecular Sciences, Queen's University, Kingston, ON, Canada; Biomedical and Molecular Sciences, Queen's University, Kingston, ON, Canada; Biomedical and Molecular Sciences, Queen's University, Kingston, ON, Canada

## Abstract

**Background:**

An impairment of vagally-mediated satiety signalling has been implicated in the caloric imbalance that leads to weight gain during obesity. Previous studies have suggested that a reduction in the excitability of vagal afferent neurons with cell bodies in nodose ganglia (NG) was responsible, but the cellular mechanisms are unclear. Host and bacterially derived mediators present in the small intestine and stool provide a physiologically relevant model to help elucidate the role luminal mediators play in modulating vagal afferent neuronal excitability.

**Aims:**

We hypothesize that the microbiota of obese individuals and mice produce mediators that impair NG neuron excitability and satiety in mice.

**Methods:**

Perforated patch clamp was used to measure the excitability of NG neurons following exposure to human and mouse fecal supernatants (FS), mouse jejunal supernatants (JS), and mice serum samples. Human FS were from ampersand:003E 5 healthy human donors or FS from ampersand:003E 5 obese donors. Mice FS, JS and serum samples were collected from ampersand:003E 5 obese mice fed a high-fat diet and ampersand:003E 5 control mice fed a normal diet.

**Results:**

NG neurons incubated in FS from obese participants were significantly less excitable (rheobase was 30% higher and action potential discharge at 2x rheobase was 50% lower) than NG neurons exposed to FS from non-obese participants. NG neurons incubated in FS or JS from obese mice were also significantly less excitable (rheobase was 65% higher and action potential discharge at 2x rheobase was 50% lower) than NG neurons incubated with FS or JS from control mice. Lastly, NG neurons incubated with obese mouse serum were significantly less excitable (rheobase was 50% higher and action potential discharge at 2x rheobase was 80% lower) than NG neurons incubated with serum from control mice.

We then attempted to identify mediators that may account for this inhibitory effect by using receptor antagonists that block GABA, ghrelin, and orexin signalling. Ghrelin and GABA receptor antagonists did not block the inhibitory effect of obese patients’ FS on NG neurons but the orexin receptor 1 antagonist (*SB-334867*;10µM) did. Following this, we incubated the orexin receptor 1 antagonist (*SB-334867*;10µM) on NG neurons incubated with mouse FS and JS and observed a similar blocking of inhibitory effects back to control values.

**Conclusions:**

These findings suggest that the gut luminal contents of obese mice and humans contain an orexin receptor agonist that inhibits satiety and may contribute to over-eating.

**Funding Agencies:**

CIHRNSERC

